# Lysophosphatidic acid enhances human umbilical cord mesenchymal stem cell viability without differentiation via LPA receptor mediating manner

**DOI:** 10.1007/s10495-017-1399-6

**Published:** 2017-08-01

**Authors:** Narengerile Li, Ya-Li Yan, Sachaofu Fu, Rui-Juan Li, Peng-Fei Zhao, Xi-Yuan Xu, Jing-Ping Yang, Alatangaole Damirin

**Affiliations:** 1 Department of Biology, College of Life Sciences, Inner Mongolia University, Hohhot, 010021 Inner Mongolia China; 2 Department of Respiratory and Critical Medicine, The Third Affiliated Hospital, Inner Mongolia Medical University, Baotou, 014010 Inner Mongolia China

**Keywords:** Lysophosphatidic acid, Human umbilical cord mesenchymal stem cells, Cell survival, Cell-surface marker, Receptor mediated signaling

## Abstract

Human umbilical cord mesenchymal stem cells (hUC-MSCs) are potential stromal cells which are regarded as the most feasible stem cell group in cell therapy. The maintenance of cell survival without differentiation is important in cell transplantation and stem cell therapy. However, negative factors exist in cell transplantation. Lysophosphatidic acid (LPA) is a non-antigenic small molecule phospholipid which induced several fundamental cellular responses, such as cell proliferation, apoptosis and migration. In this study we aimed to explore the effects of LPA on the survival and differentiation of MSCs and its availability in cell therapy. We found that LPA stimulated hUC-MSC proliferation and protected hUC-MSCs from lipopolysaccharide (LPS) induced apoptosis. We also observed that CD29, CD44, CD73, CD90 and CD105 were expressed, whereas CD34 and CD45 were not expressed in hUC-MSCs, and these makers have no change in LPA containing medium, which indicated that LPA accelerated the survival of hUC-MSCs in an undifferentiating status. We also demonstrated that higher expressed LPAR1 involved in LPA stimulated cell survival action. LPA stimulated cell proliferation was associated with LPAR1 mediated G_i/o_-proteins/ERK1/2 pathway. On the other hand, LPA protected hUC-MSCs from LPS-induced apoptosis through suppressing caspase-3 activation by LPAR1 coupled with a G protein, but not G_i/o_ or G_q/11_ in hUC-MSC. Collectively, this study demonstrated that LPA increased the proliferation and survival of hUC-MSCs without differentiation through LPAR1 mediated manner. Our findings provide that LPA as a anti-apoptotic agent having potential application prospect in cell transplantation and stem cell therapy.

**Electronic supplementary material:**

The online version of this article (doi:10.1007/s10495-017-1399-6) contains supplementary material, which is available to authorized users.

## Introduction

The maintenance and repair of adult tissues and organ are guaranteed by the adult stem cell pool. Among adult stem cells, mesenchymal stem cells (MSCs) are emerging as hopeful candidates for cell-based therapy of numerous diseases [1]. As a class of multi-potent adult stromal cell, MSCs are capable of self-renewal and multi-lineage differentiation into various tissues of mesodermal origin [2, 3]. It was shown that MSCs not only engraft and differentiate into cardiomyocytes, but also stimulate the proliferation and differentiation of endogenous cardiac stem cells, and the proliferation of endothelial progenitor cells, improving normal endothelial function [4]. After in vitro culture expansion, MSCs are characterized by their capability to adhere to plastic, develop as fibroblast colony-forming-units, and differentiate into osteocytes, chondrocytes, and adipocytes, which are positive for CD73, CD90, and CD105 and negative for CD11b, CD14, CD34, CD45, and HLA-DR [5, 6]. MSCs can be easily isolated and expanded ex vivo from a wide range of adult and perinatal tissue such as the cord blood and Wharton jelly of the umbilical cord. In particular, the human umbilical cord mesenchymal stem cells (hUC-MSCs) are noninvasive and non-hematopoietic cells without ethical concerns when the cord is clamped [7]. The cell therapy protocols generally require hundreds of million hMSCs per treatment and, consequently, these cells need to be expanded in vitro for about 10 weeks before implantation [1]. However, hUC-MSCs,as all cultured primary cells, do not grown infinitely, but undergo only a limited number of cell division [8], and easily differentiate in vitro condition. The more important fact is that 5–25% of all injected stem cells are apoptotic [9].

Our previous study showed that lipoprotein-associated Lysophosphatidic acid (LPA) plays an important role in DNA synthesis and migration in human coronary artery smooth muscle cells [10, 11]. LPA is a small glycerophospholipid (molecular weight: 430–480 Da) which is present in all eukaryotic tissues at low concentrations and can be detected in high nanomolar to low micromolar concentrations in the blood and lymph [12–15]. As one of the lipids, LPA performs several major functions in the body. As an intercellular signalling molecule, LPA directly exerts functions through cognate G protein–coupled receptors (GPCRs) and intracellular second messenger systems of individual cells and tissues [16–18]. LPA is involved in the regulation of fundamental cellular functions including cell proliferation, differentiation, migration, adhesion, morphogenesis, cytoskeletal changes and calcium influx [19–22]. Moreover, LPA plays a fundamental role in normal physiological processes and diseases, including nervous system functions, vascular development, immune system functions, cancer progression, reproduction, fibrosis, obesity and gastrointestinal and cardiovascular systems functions [23, 24].

Most biological functions of LPA are mediated by specific interactions with its five or more cognate receptors, known as LPA receptors (LPARs), on plasma membranes [25–29]. LPAR1/Edg-2, LPAR2/Edg-4 and LPAR3/Edg-7 belong to the endothelial differentiation gene (Edg) family of receptors and present about 55% sequence identity [12, 25, 26]. LPAR4-6 is structurally distinct from Edg receptors sharing significant homology and is closely related to P2Y purinergic receptors. These receptors, which consist of seven putative transmembrane domains, couple with and activate various G proteins, including G_αi/o_, G_αq/11_, G_α12/13_ and G_αs_ [23, 30, 31]. Given the heterogeneity of receptor subtypes, expression patterns and effector pathways, the regulatory effects of LPA on biological processes are diverse and widespread. The initial foci of LPAR studies were on the vascular or nervous systems. However, these receptors also show applications in stem cell biology. In the current study, we detected the expression of LPA receptors in hUC-MSCs, explored the effects of LPA on the survival and differentiation of hUC-MSCs and elucidated the underlying mechanisms.

## Materials and methods

### Materials

1-Oleoyl-sn-glycero-3-phosphate (LPA), fatty acid-free BSA, pertussis toxin (PTX), PDGF, Ki-16425 and PD98059 were obtained from Sigma-Aldrich Co. LLC. MK-2206 and SB203580 were purchased from Selleck Chemicals. The p-ERK1/2 and ERK1/2 antibodies were purchased from Cell Signaling. YM-254890 was generously provide by Prof. Fumikazu Okajima (Gunma University, Japan). The sources of all other reagents were described in supporting information for detailed descriptions.

### Isolation and culture of hUC-MSCs

Human umbilical cords were supplied from volunteers of the Third Affiliated Hospital of Inner Mongolia Medical University. Fresh umbilical cords were washed with phosphate-buffered saline (PBS). Blood vessels were removed before cutting up into gruel. Small pieces of tissues were arranged from the inside of a culture bottle with 10 mL of Dulbecco’s modified Eagle’s medium (DMEM-F12, Gibco) supplemented with 10% (v/v) foetal bovine serum (HyClone), 0.5 ng/mL human epidermal growth factor (Promega), 2 ng/mL human fibroblast growth factor-2 (Promega) and 100 units/mL penicillin/streptomycin in a humidified (air/CO_2_ 19:1) atmosphere for 4 h at 37 °C. The medium was replaced with 15 mL of new medium after 7 days; The medium was replaced every 7 days. Adherent cells were identified by flow cytometry to test the specific cell surface markers of MSCs. Cells with four to eight passages were used in the present study and switched to serum-free medium containing 0.1% (w/v) fatty-acid-free bovine serum albumin (BSA) at 12 h before the experiments.

### Quantitative RT-PCR analysis

The total RNA was extracted from hUC-MSCs by using TRIzol reagent (Takara). One microgram of the total RNA was used to reverse transcription reaction by using PrimeScript^®^ RT reagent Kit after removing the genomic DNA through gDNA Eraser (Perfect Real Time, Takara). Quantitative real-time PCR was performed with SYBR^®^ Premix Ex Taq^TM^ II (Tli RNaseH Plus, Takara) in LightCycler^®^ 480 (Roche). The expression level of the target mRNA was normalised to the relative ratio of the expression of GAPDH and beta-actin. Each RT-PCR assay was conducted following the manufacturer’s instructions and applied at least thrice. The primers used for qPCR are listed in Online Resource 1, which are synthesized by Sangon Biotech (Shanghai).

### Cell viability assay

Cell viability was tested by TransDetect TM Cell Counting Kit (CCK, TransGen Biotech) and cell proliferation ELISA kit (BrdU, colorimetric, Roche). Cells were seeded in 24-well plates and stimulated with different reagents for 24 h. CCK was added to each well at a final concentration of 10% and then incubated for 2 h. Quantitative colorimetric assay at 450 nm was performed on a 96-well scanning spectrophotometer.

For BrdU assay, cells were seeded in 96-well plates with a final volume of 100 μL/well. After an appropriate incubation time, the assay was performed following the manufacturer’s instructions. Cells were added to 10 μL/well BrdU labelling solution and then re-incubated for 2 h at 37 °C. Two-hundred μL/well FixDenat was added to the cells after removal of labelling medium and then incubated for 30 min at +15 to +25 °C. After removal of FixDenat solution, 100 μL/well anti-BrdU-POD working solution was added to the plates, and the mixture was incubated for 90 min at +15 to +25 °C. Antibody conjugate was removed, and the wells were rinsed thrice with 200 μL/well washing solution (1× PBS). After removal of the washing solution, 100 μL/well substrate solution was added, and the mixture was incubated for 8 min at +15 to +25 °C. Twenty-five μL/well 1 M H_2_SO_4_ was then added, and the mixture was incubated for 1 min on a shaker at 300 rpm. The absorbance of the samples was measured immediately at 450 nm (reference wavelength: 690 nm) by Epoch (BioTek, USA).

### Transfection of small interfering RNA

The cells were plated on 24 multiplates at 2 × 10^5^ cells/well. Small interfering RNAs (siRNAs, 50 nM) were introduced into cells using Lipofectamine 2000 reagent (Invitrogen) according to the manufacturer’s instructions. The total RNA was extracted from cells after cultured for 24 h. The mRNA levels of LPAR1 and S1PR1 were measured using real-time SYBR Green technology. For the BrdU assay, cells were plated on 96 multiplates at 2 × 10^5^ cells/well and transfected with 50 nM siRNAs for 48 h after cell attachment. Cells were stimulated with different reagents for 24 h and detected by BrdU kit after serum starvation as described. The nonsilencing siRNA (NC) and siRNA specific to LPAR1 (M-003952-00) were obtained from Sangon Biotech (Shanghai).

### Caspase-activity assay

For lipopolysaccharide (LPS, Biotopped) induced apoptotic experiment, the cells were exposed to different conditions in 10% FBS medium, which contains LPS binding protein.

The cells were seeded in 96-well plates with a final volume of 100 μL/well. After an appropriate incubation time, the assay was performed following the manufacturer’s instructions. Apo-ONE^®^ Homogeneous Caspase-3(7)/8/9 Reagent (Promega) was added at 100 μL/well, and the mixture was incubated for 2.5 h at +15 to +25 °C (1 min on a shaker at 300 rpm). The absorbance of the samples was measured at an excitation wavelength of 485 nm and an emission wavelength of 530 nm by Varioskan Flash (Thermo Scientific, USA).

### Assessment of morphological changes by fluorescence microscopy

Chromosomal condensation was assessed using the chromatin dye Hoechst33342 (Beyotime Institute of Biotechnology, China). HUC-MSCs were fixed for 10 min in PBS containing 1% paraformaldehyde at +15 to +25 °C. After fixing, the cells were washed twice with PBS and then exposed to 10 μg/mL Hoechst 33342 in PBS for 30 min at +15 to +25 °C. All samples were observed under a fluorescence microscope. Apoptotic cells were characterised by morphological alterations such as condensed nuclei and cell shrinkage.

### Flow cytometry analysis of cell surface markers

Approximately 2 × 10^5^ cells were seeded in six-well plates for 24 h. Flow cytometry was used to detect cell surface markers after LPA stimulation for 72 h. The cells were collected and washed with PBS containing 0.5% BSA (FACS buffer) and then incubated with 20 μL each of CD34-PE, CD90-FITC, CD29-APC, CD105-PerCP, CD44-PE, CD45-FITC, CD73-PE and CD71-FITC mouse anti-human monoclonal antibody (Becton Dickinson, USA) in a total volume of 100 μL for 20 min at +15 to +25 °C in dark place. The cells were pelleted and then washed thrice in FACS buffer. The cells were then fixed with 2% paraformaldehyde in FACS buffer and subjected to flow cytometry on a FACScan flow cytometer (Becton Dickinson, USA).

### Western blot assay for ERK1/2 activation

HUC-MSCs were seeded in 60 mm dish and then starved for 6 h in serum-free medium. The cells were washed twice with ice-cold PBS and then lysed with ice-cold RIPA buffer (Solarbio, China). The cell lysate was centrifuged at 14,000×*g* for 10 min at 4 °C and the protein concentration was determined by the BCA assay. Equal amounts of protein were separated on 10% SDS-PAGE gels by electrophoresis, then transferred to PVDF membranes using semi-dry electroblotting apparatus. The membranes were blocked for 2 h at room temperature in 5% skim milk, then incubated with primary antibody in 2% skim milk over night at 4 °C and secondary antibody in 5% skim milk for 2 h at room temperature.

### Data analysis

The mean values of the different groups were showed as mean ± SD. All statistical comparisons were performed using one-way ANOVA followed by Bonferroni’s post-hoc test for multi-group comparisons in GraphPad Prism version 5.01 (GraphPad Software, San Diego, CA). Statistical significance was assessed by Student’s *t* test, and the values were considered significant at p < 0.05. Statistical significance is designated as asterisk in the figure legends.

## Results

### LPA enhanced hUC-MSCs survival through proliferation and anti-apoptotic action

**Fig. 1:**
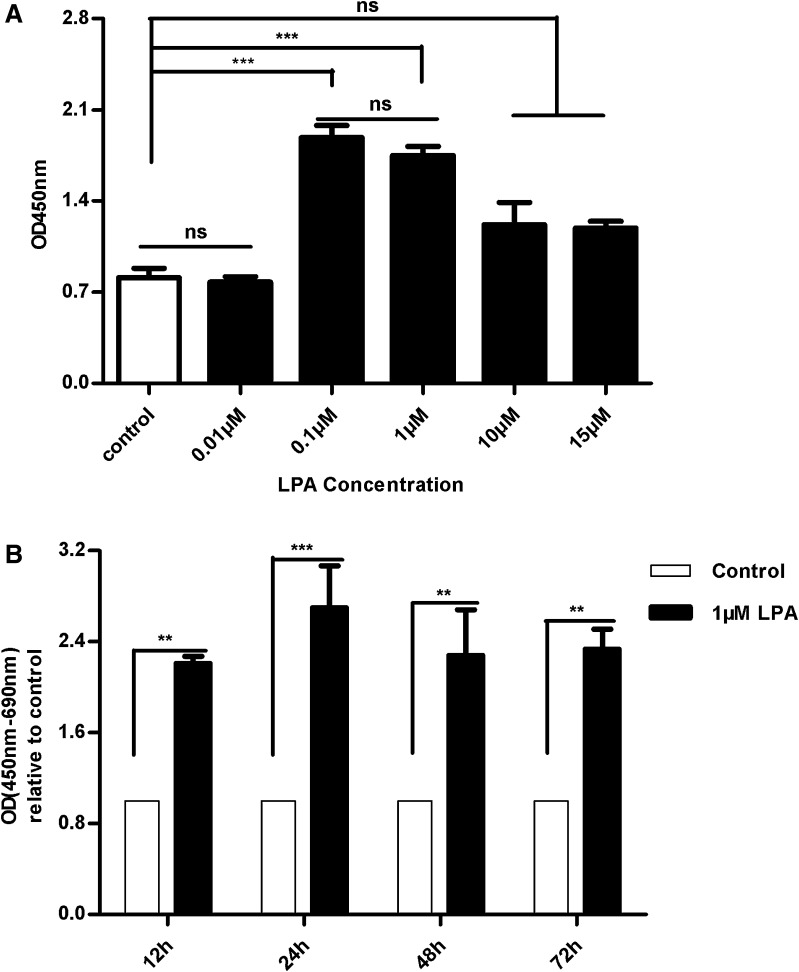
LPA increased the proliferation of hUC-MSCs. **a** CCK kit was used to determine the cells proliferation after LPA treated with a various concentrations (0.01, 0.1, 1, 10 and 15 μM) for 24 h. **b** HUC-MSCs in 96-multiplates were stimulated with 1 μM LPA for different time, then measured by BrdU assay. Absorbance was read at 450 nm (690 nm as reference) to determine cell proliferation. The *asterisk* (*) indicates that the effects were significant. Values are means ± SD and represent three independent experiments (*p ≤ 0.05, **p ≤ 0.01 and ***p ≤ 0.001)

To explore the availability of LPA in cell therapy, we first examined the ability of LPA to stimulate the proliferation of hUC-MSCs by CCK kit. As shown in Fig. 1a, cell numbers were increased by LPA stimulation in a dose-dependent manner with a peak at 0.1–1 μM. Meanwhile, we also found that LPA induced a significantly increasing DNA synthesis since 12 h and a peak at 24 h, when detected by BrdU assay (Fig. 1b).

**Fig. 2:**
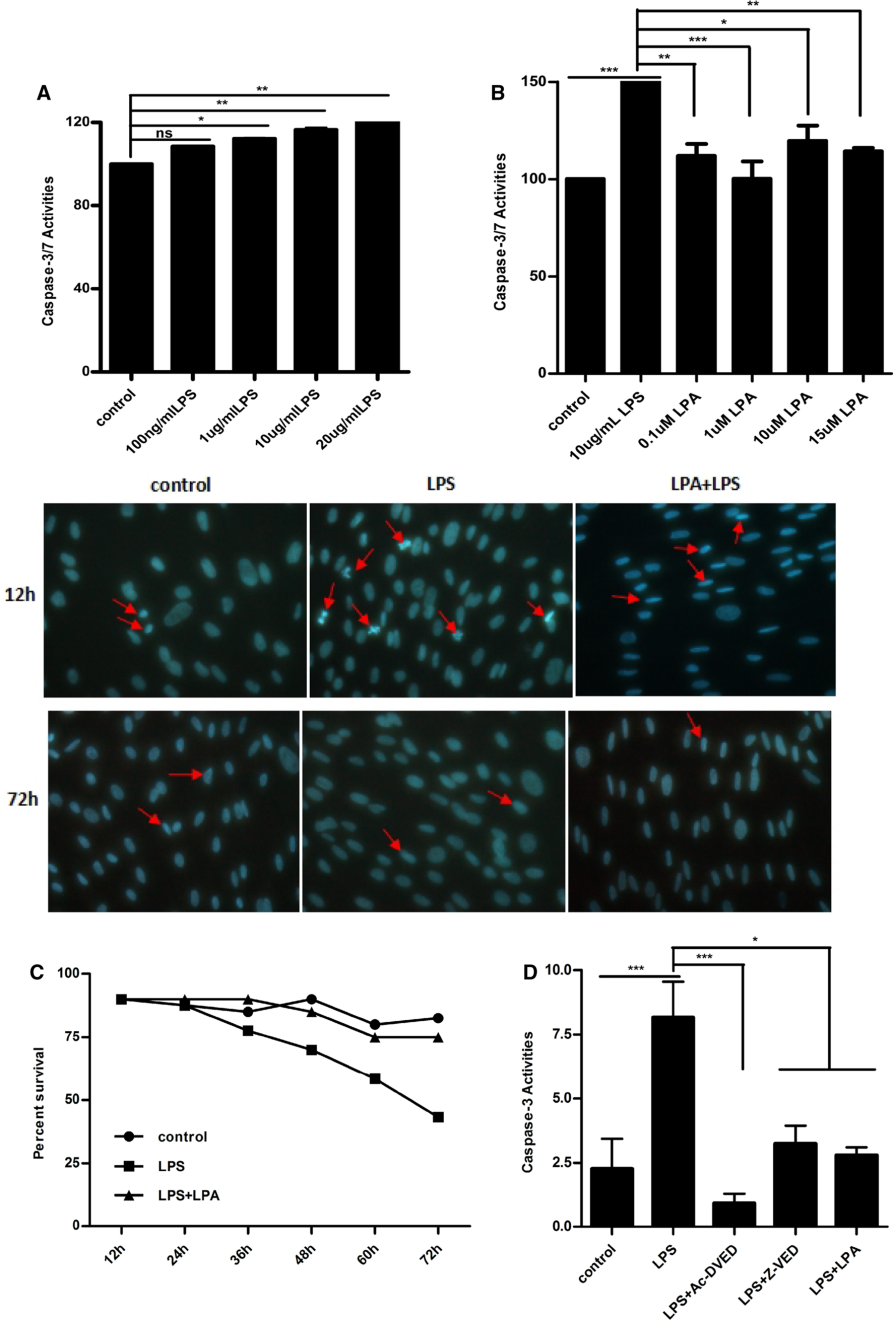
LPA protected hUC-MSCs from LPS-induced apoptosis. **a** HUC-MSCs in 96-multiplates were incubated with various concentrations of LPS for 24 h in an antibiotic-free culture medium with 10% FBS. The caspase-3 activity was examined by Apo-ONE^®^ Homogeneous caspase-3/7 Assay. **b** The cells were incubated with various concentrations of LPA and 10 μg/mL LPS for 48 h and the caspase-3/7 activity was detected. **c** HUC-MSCs in 24-multiplates were incubated with 1 μM LPA and 10 μg/mL LPS at indicated time, and stained in 10 μg/mL Hoechst 33342 in PBS for 30 min. After fixing with 1% paraformaldehyde for 10 min. The images of cells were captured using a fluorescence microscope. **d** HUC-MSCs in 96-multiplates were stimulated with different condition (10 μg/mL LPS, 1 µM LPA, 100 nM caspase-3 inhibitor Ac-DEVD-CHO and 10 µM caspase inhibitor Z-VAD-FMK) for 24 h in antibiotic free culture medium included 10% FBS. The caspase-3 activity was detected by Apo-ONE^®^ homogeneous caspase-3/7 assay. Values are means ± SD and represent three independent experiments (*p ≤ 0.05, **p ≤ 0.01 and ***p ≤ 0.001)

We wondered if the increasing cell number response to LPA are due to the proliferation and anti-apoptotic action. Therefore we performed lipopolysaccharide (LPS) induced apoptosis experiments. As shown in Fig. 2a, cells were incubated with LPS (0.1, 1, 10 and 20 μg/mL) in 10% FBS containing medium for 24 h before determining caspase-3 activity, and 10 μg/mL LPS was selected as the optimum dose for the apoptotic experiment. The caspase-3 activity response to LPS was completely inhibited by LPA at 1 μM (Fig. 2b). In parallel, cell death was determined morphologically, as shown in Fig. 2c. The control cells exhibited a normal elongated MSC morphology with large regular nuclei. After LPS treatment with 1 μM LPA, clear apoptotic characteristics of shrinkage in cell size and cell loss together with clear chromatin condensation and typical fragmented nuclei were observed. LPA efficiently blocked apoptosis with cells maintaining their elongated morphology and large nuclei. Ac-DEVD-CHO and Z-VAD-FMK are the selective inhibitor of caspase-3. Therefore, we attempted to confirm the inhibition of caspase-3 activity response to LPA using Ac-DEVD-CHO and Z-VAD-FMK. As shown in Fig. 2d, LPA suppressed LPS induced caspase-3 activity y about 60% and the inhibitory effect was the same as Z-VAD-FMK. These results suggest that anti-apoptotic action in respose to LPA is mediated by suppressing caspase-3 activation in hUC-MSCs.

### LPA had no effect on the expression of cell surface markers of hUC-MSCs

**Fig. 3:**
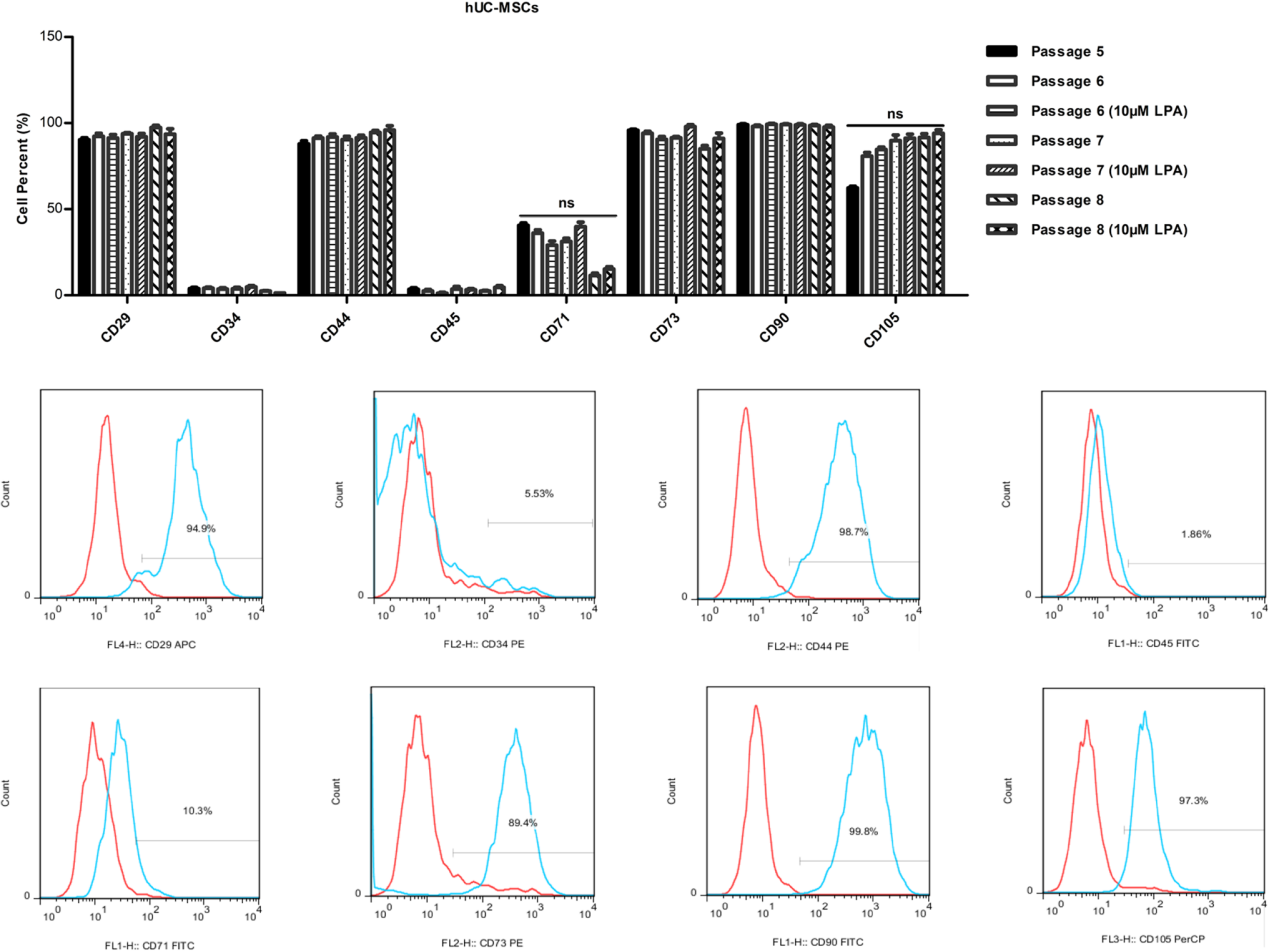
LPA had no effect on the expression of cell surface markers of hUC-MSCs. Five to eight passages serum-starved hUC-MSCs were pretreated with or without 10 μM LPA for 72 h, and the cell surface markers were detected by flow cytometry. Each sample was mixed with 20 μL of IgG1-FITC, IgG1-PE, IgG1-APC and IgG1-PerCP for the control. HUC-MSC specific surface markers (20 μL), such as CD90-FITC, CD29-APC, CD105-PerCP, CD44-PE, CD45-FITC, CD73-PE, CD34-PE and CD71-FITC were added to the samples to detect differentiation by flow cytometry. Values are means ± SD and represent three independent experiments (*p ≤ 0.05, **p ≤ 0.01 and ***p ≤ 0.001)

The International Society for Cellular Therapy (ISCT) has proposed a set of standards to define MSCs. A cell can be classified as an MSC if it shows plastic adherent properties under normal culture conditions and exhibits a fibroblast-like morphology. Cultured MSCs also express CD73, CD90 and CD105 on their surface but do not express CD11b, CD14, CD19, CD34, CD45, CD79a and HLA-DR [5]. Therefore, we investigated whether LPA can induce the differentiation of hUC-MSCs. As shown in Fig. 3 and Online Resource 2, hUC-MSCs express mRNAs of CD29, CD34, CD44, CD45, CD71, CD73, CD90 and CD105. The expression pattern of these cell surface markers have no change in hUC-MSCs cultured in with or without LPA stimulation.

### LPA stimulated hUC-MSCs proliferation was mediated by LPAR1/G_i/o_ and G_q/11_-protein/ERK1/2 pathway

**Fig. 4:**
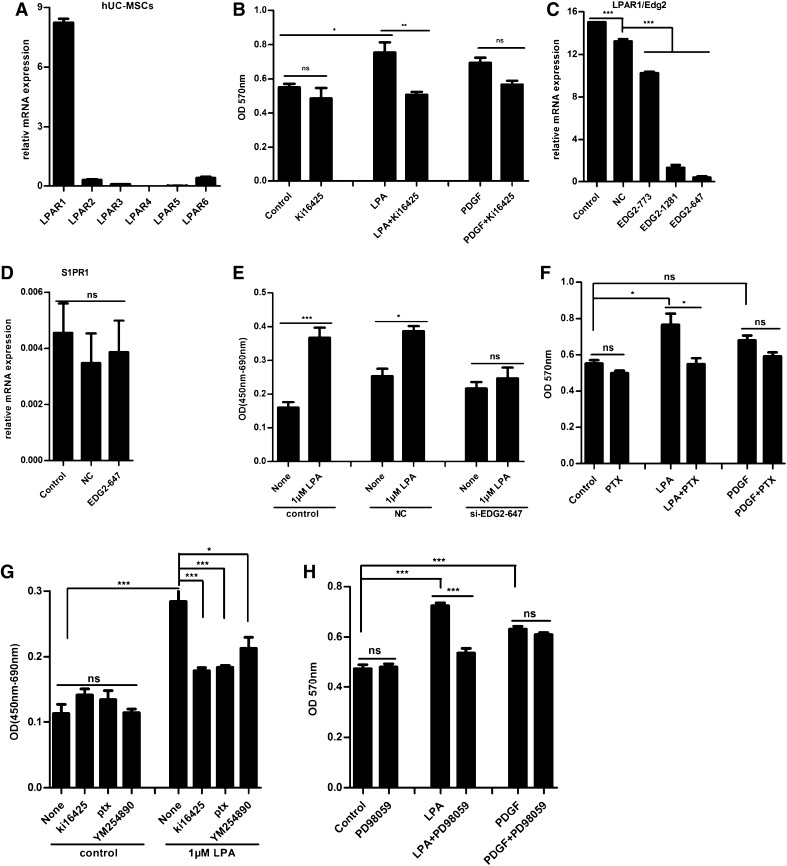
LPA enhenced proliferation of hUC-MSCs mediated by LPAR1 coupled to G_i/o_-protein. **a** mRNA expression of LPARs were detected by RT-PCR relative to GAPDH and beta-actin in hUC-MSCs. LPAR1 mRNA was profoundly higher than any other receptors. **b** The cell growth in response to 1 μM LPA was measured by MTT for 48 h with presence or absence of 10 μM Ki16425 (an LPAR1/3 antagonist) in hUC-MSCs. **c, d** HUC-MSCs in 24-multiplates were transfected with 50 nM NC siRNA (negative control) or LPAR1-specific siRNA (EDG2-homo-1281, EDG2-homo-647, EDG2-homo-773) for 24 h, then extracted total RNA. The mRNA for LPAR1 and S1PR1 in hUC-MSCs were assessed by real-time SYBR Green PCR. Results are showed as the relative ratios to GAPDH and β-actin mRNA expression. **e** HUC-MSCs in 96-multiplates were stimulated with 1 μM LPA for 24 h after transfected with 50 nM NC siRNA (negative control) or LPAR1-specific siRNA EDG2-homo-647 for 48 h. The cell proliferation was measured by BrdU assay. Absorbance was read at 450 nm (690 nm as reference) to determine cell proliferation. **f** HUC-MSCs in 24-multiplates were incubated with 10 μM LPA or 10 ng/mL PDGF for 48 h by MTT after pretreated with the indicated inhibitors. The cells were pretreated with 100 ng/mL PTX (pertussis toxin, an inhibitor of G_i/o_-protein coupled receptors) for 16 h. **g** HUC-MSCs in 96-multiplates were stimulated with 10 μM LPA for 12 h after pretreated with indicated inhibitors (10 μM Ki16425, 100 ng/mL PTX and 100 nM YM254890, a specific inhibitor of G_q/11_-proteins). The cell proliferation was measured by BrdU assay. Absorbance was read at 450 nm (690 nm as reference). **h** The cells in 24-multiplates were pretreated with 10 μM PD98059 (an inhibitor of ERK1/2) for 30 min and incubated with 10 μM LPA or 10 ng/mL PDGF for 48 h, then detected by MTT. Values are means ± SD and represent three independent experiments (*p ≤ 0.05, **p ≤ 0.01 and ***p ≤ 0.001)

In order to clarify LPA receptor subtypes involved in the proliferation response, we examined the expression patterns of LPA receptor mRNA in hUC-MSCs. Among the LPA receptor subtypes, LPAR1 mRNA is expressed at an extremely high level, supporting the possible role of LPAR1 in the LPA induced proliferation (Fig. 4a). As shown in Fig. 1c, LPA stimulated proliferation was almost completely inhibited by Ki16425, an LPAR1- and LPAR3-specific antagonist. Ki16425 did not affect the PDGF-induced proliferation, indicating the specificity of the agent (Fig. 4b). To confirm the involvement of LPAR1 in LPA induced action, siRNA specific to LPAR1 was employed. We found that the siRNA inhibited LPAR1 mRNA expression without having any significant effect on the mRNA expression of S1P1 receptor, which also belong to the Edg family of receptors (Fig. 4c, d). Under the conditions, siRNA specific to LPAR1 markedly inhibited the stimulated proliferation response to LPA (Fig. 4e). The results suggest that LPAR1 mediate the LPA-induced proliferation response in hUC-MSCs.

Based on our current results described above, we were to further research the mechanisms of LPA induced proliferation of hUC-MSCs. LPAR1 are reported to be coupled to G_i/o_-proteins and G_q/11_-proteins [18], we examined whether G-proteins involved in LPA induced proliferation by using inhibitors. As shown in Fig. 4a, LPA stimulated proliferation was almost completely inhibited by pertussis toxin (PTX), a specific inhibitor of G_i/o_-proteins. PTX did not affect the PDGF-induced action (Fig. 4f). These results suggest that LPA stimulated proliferation through G_i/o_-protein-coupled LPAR1 receptor in hUC-MSCs. By way of BrdU ELISA assay with inhibitors, we also found that YM254890, a specific inhibitor of G_q/11_-proteins, has slightly effects on the LPA action (Fig. 4g). Meanwhile, LPA stimulated proliferation was also almost completely inhibited by PD98059, an inhibitor of ERK1/2, which did not affect the PDGF-induced action (Fig. 4h).

**Fig. 5:**
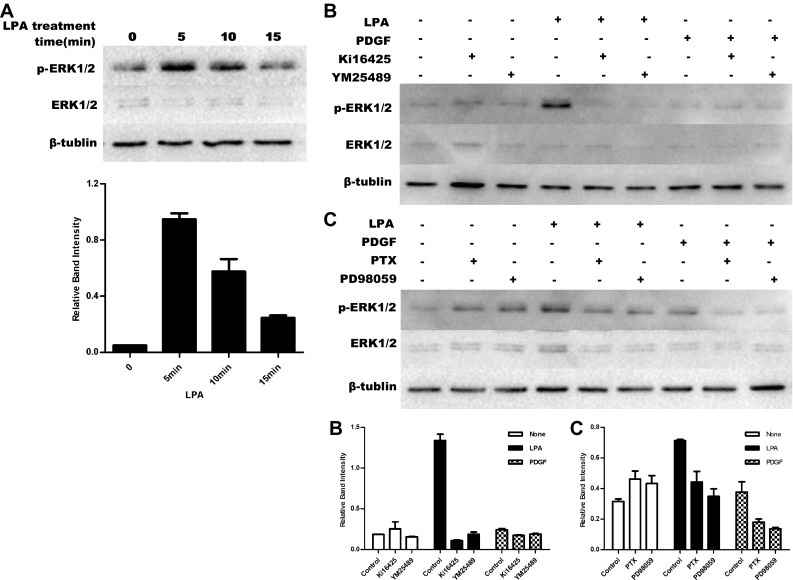
LPA stimulated the proliferation of hUC-MSCs by LPAR1 mediated G_i/o_ and G_q/11_-proteins/ERK1/2 pathway. **a** HUC-MSCs were stimulated with 10 µM LPA at the indicated time. The effects of LPA on ERK1/2 phosphorylation in hUC-MSCs were detected by western blot. **b** HUC-MSCs were stimulated with 10 µM LPA for 5 min or 10 ng/mL PDGF for 10 min after pre-treated with or without 1 μM Ki16425 for 15 min and 100 nM YM254890 for 30 min. ERK1/2 phosphorylation were detected by western blot. **c** HUC-MSCs were stimulated with 10 µM LPA for 5 min after pre-treated with 100 ng/mL PTX for 16 h or 10 μM PD98059 for 30 min. ERK1/2 phosphorylation were detected by western blot. Values are means ± SD and represent three independent experiments (*p ≤ 0.05, **p ≤ 0.01 and ***p ≤ 0.001)

LPA induced ERK1/2 phosphorylation shown a time-dependent with a peak at 5 min in hUC-MSCs (Fig. 5a). LPA induced ERK1/2 phosphorylation was inhibited by Ki16425 and YM254890 treatment (Fig. 5b). As shown in Fig. 5c, LPA stimulated ERK1/2 phosphorylation was markedly inhibited when cells were pre-treated with PTX for 16 h and PD98059 for 30 min in hUC-MSCs, suggesting that LPA/LPAR1-mediated proliferation in hUC-MSCs was dependent on PTX-sensitive G_i/o_ and G_q/11_ protein and ERK1/2 signalling pathways.

### LPA enhanced the survival of hUC-MSCs in a PTX-independent manner

**Fig. 6:**
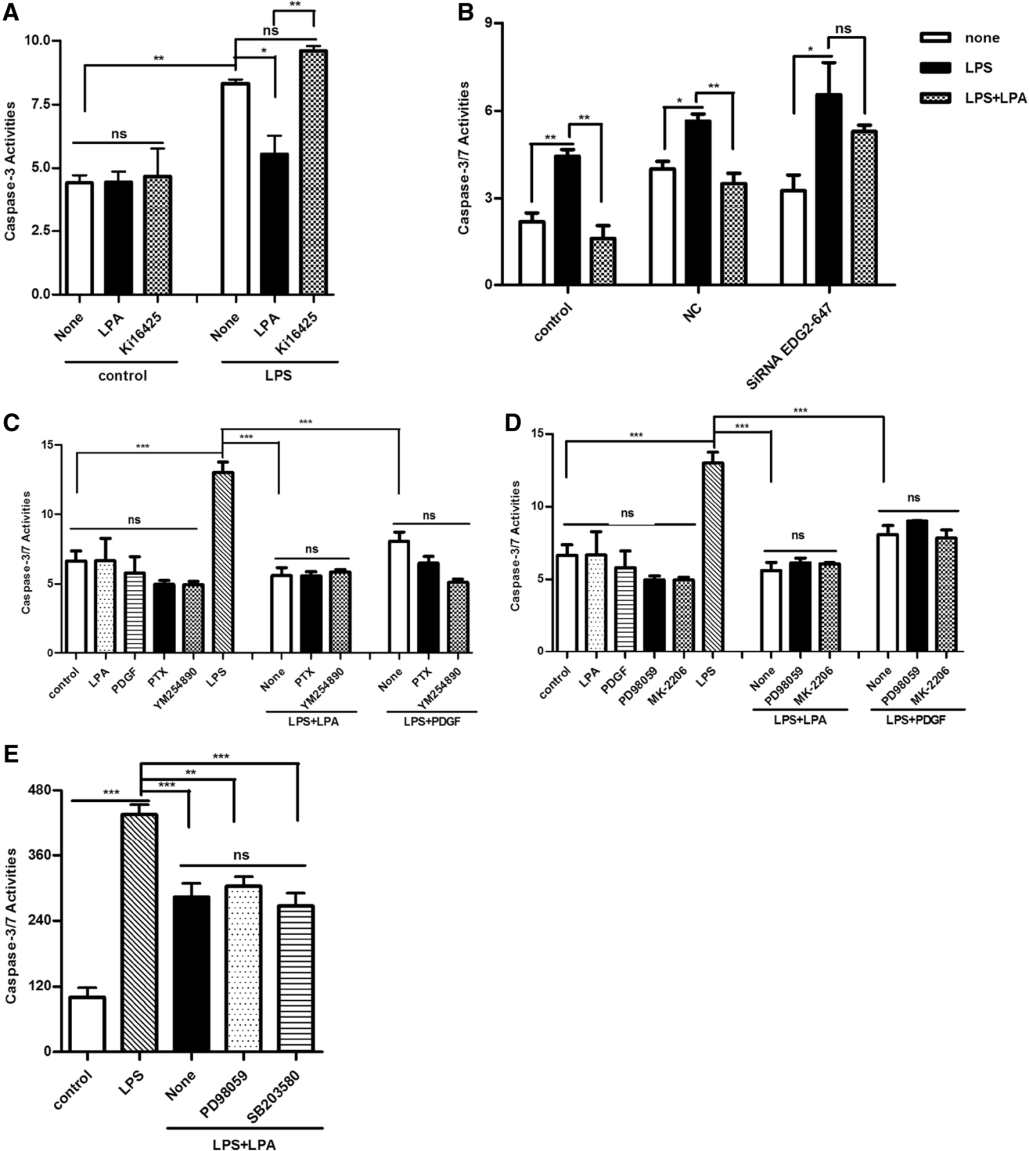
LPA enhenced the survival of hUC-MSCs in PTX-independent manner. **a** HUC-MSCs in 96-multiplates were stimulated with 1 µM LPA and 10 μg/mL LPS for 72 h after pretreated with or without 10 μM Ki16425 for 30 min. The caspase-3/7 activity was then detected. **b** HUC-MSCs in 96-multiplates were stimulated with 1 μM LPA and 10 μg/ml LPS for 24 h after transfected with 50 nM NC siRNA (negative control) or LPAR1-specific siRNA EDG2-homo-647 for 48 h. The caspase-3/7 activity was then detected. **c** HUC-MSCs were stimulated with 1 µM LPA and 10 μg/mL LPS for 24 h after pretreated with 100 ng/mL PTX for 16 h or 100 nM YM254890 for 30 min. The caspase-3 activity was examined by Apo-ONE^®^ homogeneous caspase-3/7 assay. **d** HUC-MSCs were stimulated with 1 µM LPA and 10 μg/mL LPS for 24 h after pretreated with 10 μM PD98059 or 200 nM MK-2206 (an inhibitor of Akt, Selleckchem) for 30 min. The caspase-3 activity was examined by Apo-ONE^®^ homogeneous caspase-3/7 assay. **e** HUC-MSCs in 96-multiplates were stimulated by 10 μg/mL LPS and 1 µM LPA for 24 h with or without indicated inhibitors (10 μM Erk1/2 inhibitor PD98059 and 10 μM p38 MAP kinase inhibitor SB203580). The caspase-3 activity was detected by Apo-ONE^®^ homogeneous caspase-3/7 assay. Values are means ± SD and represent three independent experiments (*p ≤ 0.05, **p ≤ 0.01 and ***p ≤ 0.001)

In order to examine the roles of LPA receptor subtypes in the anti-apoptitic action of LPA, Ki16425 was used. As shown in Fig. 6a, 10 µM Ki16425 completely reversed LPA induced inhibitory action to caspase-3 activation. SiRNA specific to LPAR1 was used to confirm the involvement of LPAR1 in the action. LPA induced anti-apoptosis was inhibited by LPAR1 siRNA, suggesting LPAR1 mediate the LPA induced anti-apoptosis in hUC-MSCs (Fig. 6b). The further researches about the mechanism of LPA suppressed caspase-3 activity were employed with inhibitors. We found PTX (100 ng/mL) and YM254890 (100 nM) have no effect on LPA suppressed caspase-3 activity (Fig. 6b). Meanwhile, we also found that all inhibitors of PD98059 (an inhibitor of ERK1/2), MK-2206 (an inhibitor of Akt, Selleckchem) and SB203580 (an inhibitor of p38 MAP kinase) have no effect on LPA suppressed caspase-3 activity (Fig. 6c, d). These data indicated that LPA remarkably increased hUC-MSC survival through LPAR1/3 in a PTX-independent manner.

### LPA elicited anti-apoptotic effect by inhibiting caspase-3

**Fig. 7:**
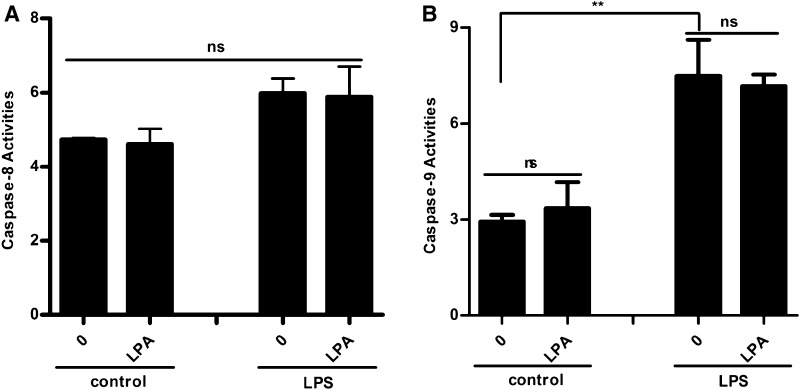
LPA elicited anti-apoptotic effect by inhibiting caspase-3. **a** HUC-MSCs in 96-multiplates were incubated with 1 μM LPA and 10 μg/mL LPS for 48 h. The caspase-8 activity was detected by Apo-ONE^®^ homogeneous caspase-8 assay. **b** HUC-MSCs in 96-multiplates were incubated with 1 μM LPA and 10 μg/mL LPS for 48 h. The caspase-9 activity was detected by Apo-ONE^®^ homogeneous caspase-9 assay. Values are means ± SD and represent three independent experiments (*p ≤ 0.05, **p ≤ 0.01 and ***p ≤ 0.001)

LPA promoted hUC-MSC survival by decreasing the activation of caspase-3. We finally examined both caspase-8 and caspase-9 to determine which apoptotic signalling pathway is involved in the LPA-induced reduction of activated caspase-3. As shown in Fig. 7a, b, caspase-9 activity significantly increased after LPS-induced apoptosis with decreasing caspase-8 activity. Interestingly, LPA did not affect caspase-9 activity. These results indicated that LPS induced apoptosis through the Fas (cytochrome C/caspase-9/caspase-3) pathway and that LPA decreased the activation of caspase-3 through LPAR1/3 in a PTX-independent manner to promote hUC-MSC survival.

## Discussion

HUC-MSCs are potential stromal cells isolated from the umbilical cord and are regarded as the most feasible stem cell source for cell therapy. Umbilical cords can provide inexhaustible and inexpensive stem cell sources without invasive surgery and controversies [32]. HUC-MSCs are primitive MSCs which exhibit a high plasticity and developmental flexibility [33]. In the current study, hUC-MSCs were isolated effectively and used to detect cell surface markers by flow cytometry. However, hUC-MSCs and other stem cell applications still have several negative characteristics, such as easily differentiation or low survival. Therefore, to study on the development of bio-active reagents in order to improve stem cell survivability and maintain the cell keeping in undifferentiated state is very important, which have being a focus in field of cell therapy.

LPA, a pleiotropic lipid growth factor, induces many fundamental cellular responses, such as cell proliferation, apoptosis and migration through combination with GPCR LPARl-6. In our previous study, we found that LPA/LPA1 receptors mediate the DNA synthesis and migration response to LDL in coronary artery vascular smooth muscle cells [10, 11] and LPA/LPA2 receptors stimulates the migration of human gastric cancer cells (SGC-7901) [34]. LPA is a bioactive endogenous phospholipid without immunogenicity. LPARs are expressed in many types of cells, demonstrating a differential expression profile across various cells and tissues [35, 36]. LPA is also a proliferative survival factor in various stem cells. In the current study, we assessed the effect of LPA on the survival and differentiation of hUC-MSCs and its availability in cell therapy. We found that LPA stimulated hUC-MSC proliferation in a dose-dependent manner. Furthermore, we examined the inhibitory effects of LPA on LPS-induced hUC-MSC apoptosis. We found that LPA protected hUC-MSCs from LPS-induced apoptosis. The maintenance of cell survival without differentiation is essential in stem cell therapy. Thus, we examined the effects of LPA on the differentiation of hUC-MSCs through detection of cell surface markers. Flow cytometry demonstrated that CD29, CD44, CD73, CD90 and CD105 were expressed in hUC-MSCs, whereas CD34 and CD45 were not expressed. The results accord with the standards of MSCs proposed by ISCT. Our results showed that hUC-MSCs were maintained undifferentiated stage for eight passages at LPA stimulated condition, suggesting LPA accelerated the proliferation and survival of hUC-MSCs without differentiation. Other investigations also has been reported that LPA is capable of preventing cell apoptosis of human neural progenitor cells [37], hippocampal progenitor cells H19-7 [38], mouse embryonic stem cell [39], human CD34^+^ cells [40] and human bone marrow-derived mesenchymal stromal cells [41]. LPA rescues H_2_O_2_-induced apoptosis mainly mediated by LPAR3 interacting with G_i/o_ protein and inhibition caspase-3 cleavage in bone marrow-derived mesenchymal stem cells [42].

We examined the expression patterns of LPA receptor mRNAs in hUC-MSCs. Among theses receptors LPAR1 mRNA expression level was higher than any other receptors, supporting the possible role of LPAR1 in LPA induced proliferation and anti-apoptotic effect. To confirm the involvement of LPAR1 in LPA induced action, siRNA specific to LPAR1 and Ki16425 (LPAR1/3 antagonist) weres employed. The LPA induced proliferation and anti-apoptotic action were completely inhibited by adding Ki16425. LPAR1-specific siRNA significantly suppressed the proliferation and anti-apoptosis response to LPA, suggesting that the effects of LPA on hUC-MSCs survival were mainly mediated by LPAR1.

LPA receptor subtypes are coupled to specific G protein and transfer various downstream cell signalling pathways when activated by LPA, such as PI3K-to-Akt/PKB (G_i/o_), Ras-to-ERK (G_i/o_, G_q/11_) Rho (G_12/13_), PLC-to-PKC (G_q/11_) [20, 31, 43–45], Hippo-YAP pathway [46] and β-catenin pathway [47, 48] for pluripotency, cell survival, proliferation, differentiation/specification and migration. In our study, we found that PTX and PD98059, which are inhibitors of G_i/o_-protein and ERK1/2, respectively, completely inhibited the LPA-induced proliferation of hUC-MSCs. YM254890, a specific inhibitor of G_q/11_-proteins, has slightly effects on the LPA action. Meanwhile, LPA induced an increasing ERK1/2 phosphorylation, which was also significantly inhibited by pre-treatment of Ki16425, PTX, YM254890 and PD98059. These results showed that LPA stimulated the proliferation of hUC-MSCs through LPAR1/G_i/o_ and G_q/11_/ERK1/2 pathway. Taken together, LPA suppressed LPS-induced caspase-3 activation, as returned by Ki16425 but not by PTX and YM254890. Interestingly, the caspase-9 activity was increased by LPS significantly, but the caspase-8 was hardly affected, then the both of caspases were not affected by LPA. Overall, these results indicated that LPA shown a anti-apoptosis action through decreasing the activation of caspase-3 mediated by LPAR1 coupled with G proteins, but not G_i/o_ or G_q/11_ in hUC-MSC.

## Conclusion

**Fig. 8:**
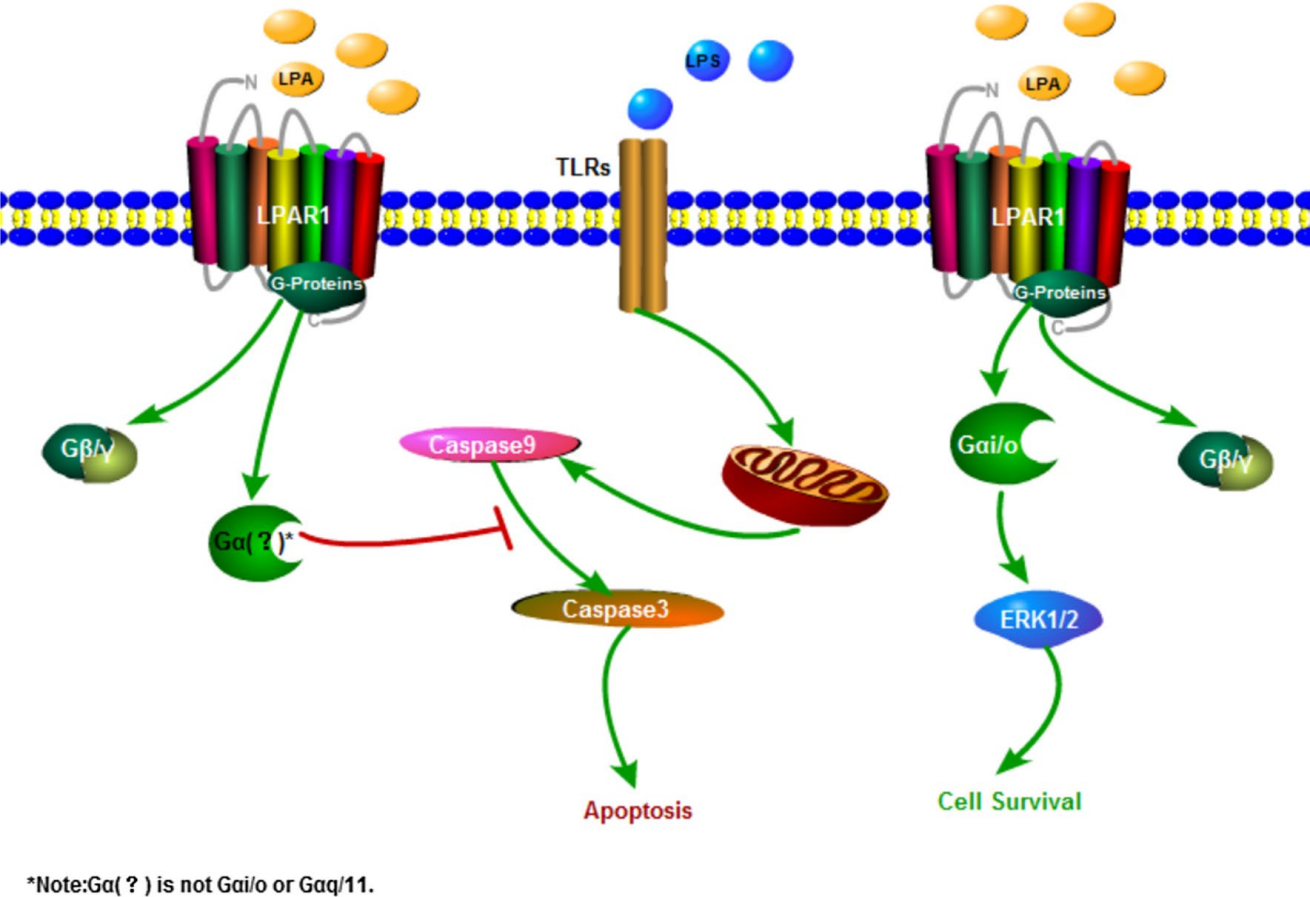
Conclusion pigture. LPA stimulated the proliferation of hUC-MSCs through LPAR1/Gi/o and Gq/11/ERK1/2 pathway. LPA shown a anti-apoptosis action through LPAR1 coupled with Gx (not Gi/o or Gq/11) mediating inhibition of caspase-3 activation in hUC-MSC

LPA stimulated the proliferation of hUC-MSCs through LPAR1/G_i/o_ and G_q/11_/ERK1/2 pathway. LPA shown a anti-apoptosis action through LPAR1 mediated inhibition of caspase-3 activation. Our results also shown that the G proteins coupled with LPAR1 were not G_i/o_ or G_q/11_ in hUC-MSC (Fig. 8). More importantly, LPA have not induce the differentiation of hUC-MSC during expansion in vitro, which is required in cell therapy and tissue engineering.

## Electronic supplementary material

Below is the link to the electronic supplementary material.

Supplementary Table 1 Primers used in this study. (DOC 26 KB)

Supplementary Table 2 Cell surface markers of hUC-MSCs. Five to eight passages serum-starved hUC-MSCs were pretreated with or without 10 μM LPA for 72 h, and the surface markers were detected by flow cytometry. Each sample was mixed with 20 μL of IgG1-FITC, IgG1-PE, IgG1-APC and IgG1-PerCP for the control. hUC-MSC specific surface markers (20 μL), such as CD90-FITC, CD29-APC, CD105-PerCP, CD44-PE, CD45-FITC, CD73-PE, CD34-PE and CD71-FITC, were added to the samples to detect differentiation. FSC/SSC clustering method was used in flow cytometry. Values are means ± SD and represent three independent experiments (*p ≤ 0.05, **p ≤ 0.01 and ***p ≤ 0.001). (DOC 27 KB)

## Abbreviations

APS: Ammonium persulfate
CD: Cluster of differentiation
DEPC: Diethyl pyrocarbonate
Edg: Endothelial differentiation gene
EGF: Epidermal growth factor
ERK1/2: Extracellular signal-regulated kinase 1/2
FGF: Fibroblast growth factor
GPCR: G-protein coupled receptor
hUC-MSCs: Human umbilical cord mesenchymal stem cells
LPA: Lysophosphatidic acid
LPS: Lipopolysaccharide
MAPK: Mitogen activated protein kinase
MTT: 3-(4,5)-Dimethylthiahiazo(-z-y1)-3,5-diphenytetrazoliumromide
NC: Negative control
PDGF: Platelet derived growth factor
PTX: Pertussis toxin
siRNA: Small interfering RNA
TEMED: Tetramethylethylenediamine
