# Supplementary material for: Lysophosphatidic acid enhances human umbilical cord mesenchymal stem cell viability without differentiation via LPA receptor mediating manner
Source: Apoptosis. 2017 Aug 1;22(10):1296–309. doi: 10.1007/s10495-017-1399-6 (PMC5630659; doi:10.1007/s10495-017-1399-6)
Supplement: Supplementary file 1 — Supplementary Table 1 Primers used in this study. (DOC 26 KB) [file 10495_2017_1399_MOESM1_ESM.doc]

Li Narengerile et al.

**LPA Enhances hMSC Survival without Differentiation**

**Online Resources**

Online Resource 1. Primers used in this study.

| Oligo Name | Sequence(5’ to 3’) | mRNA transcript_id |
| --- | --- | --- |
| H-LAPR1-DL-F | TCCTTCGTCAGGGCCTCATT | NM_001401.3 |
| H-LAPR1-DL-R | ACCGTAATGTGCCTCTCGATT |
| H-LAPR2-DL-F | GCTTCCACCAGCCCATCTAC | NM_004720.5 |
| H-LAPR2-DL-R | TGAGGAAGAGGTAGGCCACG |
| H-LAPR3-DL-F | GCAACCTGACCAAAAAGAGGG | NM_012152.2 |
| H-LAPR3-DL-R | GCAATTCCAGCCCAGTGTG |
| H-LAPR4-DL-F | GTGGCGGTATTTCAGCCTCT | XM_005262126.3 |
| H-LAPR4-DL-R | AGACACGTTTGGAGAAGCCT |
| H-LAPR5-DL-F | ACTCGGTGGTGAGCGTGTA | NM_020400.5 |
| H-LAPR5-DL-R | GTGCAGTGCGTAGTAGGAGA |
| H-LAPR6-DL-F | CACCCGCCGTTTTTGTTCAG | NM_001162498.1 |
| H-LAPR6-DL-R | ATGTTTTCCATGTGGCTTCTGG |
| H-S1PR1-SYBR-F | TGCTGGCAAATTCAAGCGAC | NM_001400.4 |
| H-S1PR1-SYBR-R | GTCCCCTTCGTCTTTCTGGG |
| TH-GAPDH-F | CCAGGTGGTCTCCTCTGACTTC | [NM_002046.3](http://www.ncbi.nlm.nih.gov/entrez/viewer.fcgi?db=nucleotide&id=223029437) |
| TH-GAPDH-R | GTGGTCGTTGAGGGCAATG |
| DL-Beta-Actin-F | GCACTCTTCCAGCCTTCCTT | NM_001101.3 |
| DL-Beta-Actin-R | AATGCCAGGGTACATGGTGG |
