# Supplementary material for: Lysophosphatidic acid enhances human umbilical cord mesenchymal stem cell viability without differentiation via LPA receptor mediating manner
Source: Apoptosis. 2017 Aug 1;22(10):1296–309. doi: 10.1007/s10495-017-1399-6 (PMC5630659; doi:10.1007/s10495-017-1399-6)
Supplement: Supplementary file 2 — Supplementary Table 2 Cell surface markers of hUC-MSCs. Five to eight passages serum-starved hUC-MSCs were pretreated with or without 10 μM LPA for 72 h, and the surface markers were detected by flow cytometry. Each sample was mixed with 20 μL of IgG1-FITC, IgG1-PE, IgG1-APC and IgG1-PerCP for the control. hUC-MSC specific surface markers (20 μL), such as CD90-FITC, CD29-APC, CD105-PerCP, CD44-PE, CD45-FITC, CD73-PE, CD34-PE and CD71-FITC, were added to the samples to detect differentiation. FSC/SSC clustering method was used in flow cytometry. Values are means ± SD and represent three independent experiments (*p ≤ 0.05, **p ≤ 0.01 and ***p ≤ 0.001). (DOC 27 KB) [file 10495_2017_1399_MOESM2_ESM.doc]

Li Narengerile et al.

**LPA Enhances hMSC Survival without Differentiation**

**Online Resources**

Online Resource 2. Cell Surface Markers of hUC-MSCs.

| Cell Surface Markers | Cell Percent (%) | | | | | | | |
| --- | --- | --- | --- | --- | --- | --- | --- | --- |
|  | Passage 5 | Passage 6 | | Passage 7 | | Passage 8 | | |
|  | control | control | LPA | control | LPA | control | | LPA |
| CD29 | 90.32±2.01 | 92.37±3.04 | 91.31±3.40 | 93.78±1.06 | 92.02±3.40 | 97.49±2.11 | 93.59±5.26 | |
| CD34 | 3.78±1.29 | 4.12±0.97 | 3.56±1.25 | 3.73±1.34 | 4.87±1.23 | 2.38±0.62 | 1.25±0.19 | |
| CD44 | 87.92±3.04 | 91.27±2.03 | 91.94±2.98 | 90.49±3.22 | 91.45±2.54 | 94.56±1.94 | 95.99±4.32 | |
| CD45 | 3.47±1.41 | 2.49±1.43 | 1.19±0.79 | 3.56±2.55 | 3.23±0.91 | 2.42±0.73 | 4.55±1.68 | |
| CD71 | 40.57±2.35 | 36.06±3.21 | 29.06±4.15 | 31.22±3.12 | 39.78±4.51 | 11.38±2.46 | 15.14±2.44 | |
| CD73 | 95.82±1.09 | 94.02±1.97 | 90.50±3.02 | 91.44±1.31 | 97.88±2.11 | 85.09±3.20 | 91.04±5.47 | |
| CD90 | 99.02±0.84 | 98.07±1.21 | 99.33±0.79 | 99.15±0.74 | 98.90±1.04 | 98.56±1.19 | 97.63±1.79 | |
| CD105 | 62.30±1.67 | 80.79±3.78 | 84.64±2.40 | 89.81±5.62 | 91.20±4.18 | 91.74±3.53 | 94.07±3.41 | |
